# Endocrinological features of a patient with 14q microdeletion and Dubowitz phenotype

**DOI:** 10.1002/mgg3.1644

**Published:** 2021-03-31

**Authors:** Maria Elisa Amodeo, Elena Inzaghi, Annalisa Deodati, Stefano Cianfarani

**Affiliations:** ^1^ Dipartimento Pediatrico Universitario Ospedaliero IRCCS "Bambino Gesù" Children's Hospital Rome Italy; ^2^ Department of Systems Medicine Tor Vergata University Rome Italy; ^3^ Department of Women's and Children's Health Karolinska Institutet and University Hospital Stockholm Sweden

**Keywords:** delayed puberty, Dubowitz syndrome, GH therapy, growth impairment, hypopituitarism

## Abstract

**Background:**

Dubowitz syndrome (DS) is a complex and rare condition characterized by postnatal growth retardation, microcephaly, short stature, mild developmental delay, facial dysmorphism, skin eruption and bone marrow failure. Though approximately 200 cases have been described so far, no specific genetic analysis, laboratory tests or radiological exams are available to confirm the diagnosis which is still based on clinical and facial features. Although short stature is a major feature of the syndrome, no endocrine alterations have been reported so far and scant data are available about the efficacy and safety of GH treatment in these patients.

**Methods:**

A 13‐year‐old male patient was referred to our attention for short stature. Endocrinological evaluation including GH axis, adrenal and gonadal functions were assessed. aCGH was performed.

**Results:**

14q terminal microdeletion associated with Dubowitz phenotype was found. Endocrinological investigations revealed the presence of hypopituitarism which showed a satisfactory response to short‐term growth hormone therapy. The subject also started glucocorticoid replacement therapy. Disorders in pubertal progression and gonadal function were noted.

**Conclusions:**

Dubowitz syndrome (DS) includes different clinical findings variably occurring.

Subjects with a Dubowitz phenotype should be carefully monitored for endocrinological anomalies. The prompt recognition of potential life‐threatening endocrinological condition for example adrenal insufficiency is mandatory in order to start an adequate and early treatment.

## Graphical abstract

We report the endocrinological features of a male patient with 14q microdeletion and Dubowitz phenotype who showed a good response to short‐term GH therapy.

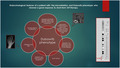

## 1 INTRODUCTION

Dubowitz syndrome (DS, % 223370) is a rare condition, firstly described in 1965, characterized by a constellation of different phenotypic features such as postnatal growth retardation, microcephaly, mild developmental delay, facial dysmorphism, bone marrow failure and propensity to malignant tumors (Wallerstein et al., 1997; Yue et al., 2013). Despite approximately 200 cases have been described so far, a specific genetic alteration has not been identified yet and the diagnosis is still made on clinical grounds. Facial features are the most recognizable signs suggestive of the syndrome (Innes et al., 2018). As currently there is no scoring system to achieve a definite diagnosis, other syndromes must be excluded in the diagnostic work‐up.

Subjects with DS often have a history of growth failure occurring both pre‐ and postnatally (Hansen et al., 1995). The affected patients may have a normal birth weight, microcephaly and develop postnatal growth retardation. Other cases have intrauterine growth restriction and severe microcephaly (Vieluf et al., 1990). A previous systematic review performed on 141 affected patients reported that 68.6% of patients had prenatal and 85.6% postnatal growth retardation (Tsukahara & Opitz, 1996). Different mechanisms have been proposed to explain the growth impairment in DS: growth hormone (GH) deficiency, gene alterations involving the GH‐IGF1 axis and disruption of specific brain structures during fetal development. However, the results of all these studies were inconclusive and the cause of the growth impairment is still unknown (Kapoor, 2015a). Scattered data indicate the possible occurrence of other endocrinological abnormalities such as early puberty described in one male patient (Tsukahara & Opitz, 1996) and hypogonadism reported in a 41 years‐old male patient (Stewart et al., 2014).

A challenging aspect refers to the DS modality of inheritance. Previous data suggested an autosomal recessive trait (Lyonnet et al., 1992; Mathieu et al., 1991; Tsukahara & Opitz, 1996). In 2018, *de novo* genetic alterations were suggested for the sporadic cases of DS (Innes et al., 2018). This conclusion was based on the recognition of genetic alterations in 21 patients with the identification of a d*e novo* genetic disorders in 14 out of 21 patients. Seven patients had *de novo* copy number variants (CNVs) in different chromosome regions such as 22q11, 14q32, 19q13, 13q, and 17q24. Seven patients had a single gene alteration with a different pattern of inheritance in LIG4 (MIM#606.593), BRCA1 (MIM#617883), PCNT (MIM#210.720), ACTB (MIM#243310), and STAT3 (MIM#615.952) genes. ACTB and STAT3 genes were *de novo* dominant variants, whereas the alterations affecting the other genes were autosomal recessive disorders with biallelic inherited variants. The remaining seven patients likely had autosomal recessive inheritance: Three siblings had biallelic variants in NSUN2 (MIM#611.091) gene and were born to consanguineous parents (Martinez et al., 2012); two siblings had biallelic variants in UBE3B (MIM#244450) gene and were born to first cousins parents; two siblings carried biallelic variant in RNU4ATAC (MIM#616.651) gene (Kukushkina et al., 1991; Mohrenschlager et al., 1998; Thuret et al., 1991). The phenotypic variability suggests the action of many genetic and/or epigenetic factors with different modalities of inheritance. Unfortunately, genetic analysis has been performed only in a minority of the reported cases. For instance, in the same review reporting the 21 genetically characterized patients with DS, the remaining 42 of 63 cases (about 66%) did not undergo any genetic analysis (Innes et al., 2018).

This case report aims at describing a child with a microdeletion 14q32.31‐q32.33 and focuses on his endocrine abnormalities and the impact of GH therapy.

## 2 CASE REPORT

**FIGURE 1:**
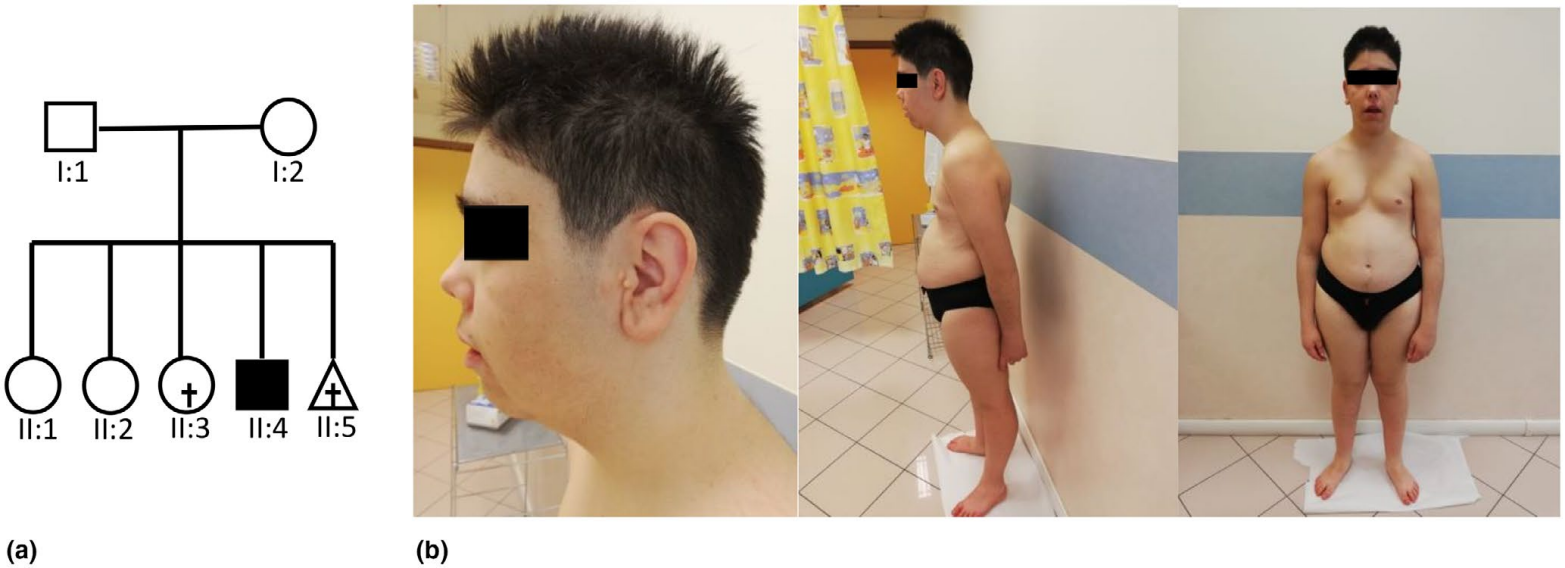
(a) Pedigree of family. (b) Photographs of the subject II‐4 at the age of 21 years

The affected boy was the fourth offspring of Italian healthy non‐consanguineous parents (Figure 1). The third female sibling died at the age of 14 years because of drug‐resistant unspecific epilepsy. Family history was characterized by autoimmune disease (father with psoriasis), sudden cardiac death (two first cousins) and thyroid disorders (mother and grandmother with chronic autoimmune thyroiditis).

The patient was born after a full‐term normal pregnancy, at 38 weeks of gestation by Cesarean section due to breech delivery. Amniocentesis, performed at the 19th week of gestation, resulted normal. Birth weight was 2.470 g (−1.99 SDS), birth length was 45 cm (−2.38 SDS), and head circumference was 32 cm (−1.96 SDS). Apgar score was not available but no neonatal health problems were reported. Audiometric and metabolic screening tests for cystic fibrosis, congenital hypothyroidism and phenylketonuria were normal. At birth, he showed facial abnormalities associated with dry eczematous skin especially on the cheeks, bilateral cryptorchidism and hoarse voice with high‐pitched tones. Additionally, moderate muscular hypotonia, sucking difficulty, regurgitation, vomiting and chronic constipation led to poor weight gain during infancy. Imaging was performed at one month of age: abdominal and cardiac ultrasound scans revealed unilateral renal agenesis with duplication of the right pielic district, polilobulated‐shape spleen and ostium secundum inter‐atrial defect, respectively. At the age of 2.5 years, cryptorchidism was surgically treated. Skeletal X‐rays performed at the age of 14 years showed severe kyphoscoliosis with D2 butterfly vertebra dysmorphism. Ophthalmologic evaluation revealed astigmatism and severe strabismus, corrected at the age of 3 years. Celiac and thyroid disease screening tests repeated at different ages were normal.

Feeding behavior and hypotonia improved with age but a global developmental delay and intellectual disability were diagnosed. He sat unsupported at 18 months, crawled at 24 months, and walked at 31 months. He showed a slow clumsy walking. Verbal capacity was poor (first words at 18 months) and persisted until adulthood, despite continuous speech training therapy. Social attitude has always been good toward family, friends and unknown individuals.

At the age of 13 years and 3 months, the patient was referred to our Endocrinology Unit for short stature. No previous anthropometric measurements were available. At the first visit, his height and weight were 136.5 cm (−2.84 SDS) and 35.5 kg (−1.83 SDS), respectively. Father's height was 165 cm with normal timing of pubertal development, and mother's height was 157.5 cm, age of menarche 12 years.

Physical examination revealed mild cognitive delay and dysmorphic facial features with high forehead, mild strabismus, epicanthal folds, blepharophimosis, palpebral ptosis, high arched palate with hypoplastic small teeth, protruding low set ears with prominent tragus/antitragus, wide lobule and asymmetrical bilateral preauricular pits and tags, upturned nose with anteverted nares, quite long philtrum, and mild micrognathia (see Figure 1, below).

Pubertal development was Tanner I stage (Marshall & Tanner, 1970) with testicular volume of 2 ml and absence of both pubic and axillary hair. Thyroid palpation was unremarkable.

Testicular ultrasonography was normal. Levels of IGF‐1, testosterone, androstenedione, dehydroepiandrosterone sulfate (DHEAS), follicle stimulating hormone (FSH), luteinizing hormone (LH), and thyroid hormones were assessed. Cortisol response to adrenocorticotropin (ACTH) stimulation and growth hormone (GH) response to stimulation tests were evaluated. Collectively, the results were indicative of normal thyroid, adrenal, and gonadal function. GH deficiency (GHD) was diagnosed (GH peaks were 2.02 and 3.16 ng/ml in response to arginine and clonidine after priming with intramuscular testosterone, respectively) with values of IGF‐1 in the low normal range (Bedogni et al., 2012) (126 ng/dl and 180 ng/dl, −2 SDS, and −1.8 SDS, respectively). Bone age, according to Greulich and Pyle method (Anderson, 1971), resulted 2 years delayed. Brain MRI showed a hypoplastic anterior pituitary gland with ectopic posterior pituitary, located in the pituitary stalk. Other findings included hypoplasia of the corpus callosum and mild asymmetry of the lateral ventricles.

The association of growth retardation with dysmorphic features and the anamnestic data of strabismus, congenital heart defects, skeletal abnormalities, cryptorchidism, eczematous skin, high‐pitched voice, and intellectual disability led us to suspect a case of DS. Array‐based comparative genomic hybridization (aCGH), using kit Human AGILENT Genome CGH Microarray Kit 44b, revealed a *de novo* microdeletion in the region 14q 32.31 q32.33 (from probe A_14_P118776 on genomic location strand 14:102180868‐102180927 to probe A_14_P135856 on genomic location strand 14:106850550‐106850609), extended for about 4.6 Mb, and confirmed by the fluorescence in situ hybridization (FISH) technique.

**FIGURE 2:**
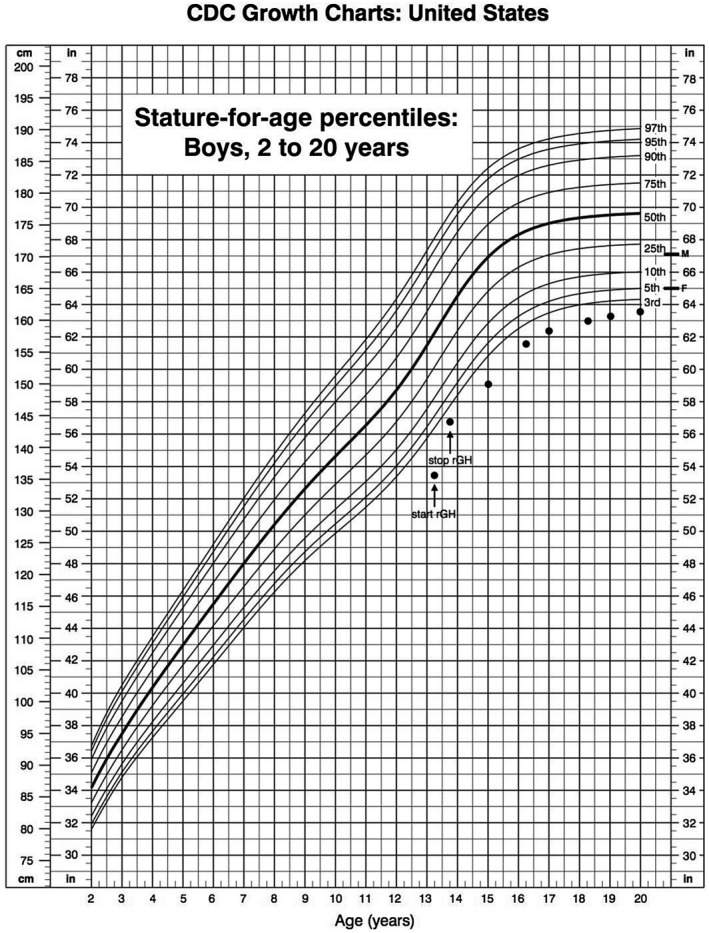
Height curve of our patient. The arrows indicate the start and the interruption of GH substitutive therapy

GH replacement therapy was started at a dose of 0.027 mg/kg/die, and first year growth velocity was 10.2 cm/year (+1.33 SDS; Figure 2). Six months after the initiation of GH therapy, routine blood exams showed a decrease in white blood cells (WBCs), red blood cells (RBCs), hemoglobin (Hb), and platelets (PLT; see Table 1). Therefore, in agreement with the family, GH therapy was stopped. During the following off‐therapy period, growth velocity initially decelerated to 4.5 cm/year (−4.13 SDS) in the first 6 months and then re‐accelerated to 7 cm/year from 14 to 16 years of age (+1 SDS) (Figure 2). Bone age was 3 years delayed at the age of 10, 2 years delayed at 13, 1.5 years delayed at 15, 1 year delayed at 16, and correspondent to chronological age at 18 years. Testicular volume was 4 ml bilaterally at the age of 15 years with testosterone levels 277 ng/dl and reached the volume of 8 ml at the age of 16. These findings were consistent with a delayed but rapid pubertal progression.

**TABLE 1:** Patient's blood cell counts before, during and after GH treatment

Hematologic indices	Before therapy	After three months of therapy	At therapy interruption	5 years after interruption
WBC [cells/mm^3^]	4460	4450	3590	3430
RBC [cells/mm^3^]	4.3 × 10^6^	4.1 × 10^6^	4.2 × 10^6^	4.1 × 10^6^
PLT [cells/mm^3^]	200	182	147	113000
Hb [g/dl]	12.6	12	12.2	12.5

The subject achieved an adult height of 160 cm (−2.5 SDS). Mid‐parental height was 167.7 cm. From the age of 15 years, IGF‐1 was annually monitored and resulted always in the normal range. The hematologic indices were monitored every 3–6 months, and the values remained permanently low, even without GH therapy (Table 1).

At the age of 21 years, the patient showed increased asthenia and sleepiness. Blood exam showed a mild thrombocytopenia and leukopenia (Table 1). An endocrinological re‐evaluation showed a low testicular volume of 12 ml bilaterally. Testicular ultrasonography showed a normal testicular structure. Hypergonadotropic hypogonadism with selective Sertoli cell impairment was suspected as basal luteotropic hormone (LH) was 10.3 mIU/ml (NV 1.7–8.6), and basal follicular stimulating hormone (FSH) was in the upper range of values of 15.4 mIU/ml (NV 1.5–12.4), without a physiological increase in testicular volume. For this reason, luteotropin‐releasing hormone (LHRH) stimulation test was performed showing a LH peak of 28 mIU/ml and FSH peakn of 21.4 mIU/ml, with testosterone levels in the low normal range (357 ng/dl, NV 249–836 ng/dl). Adrenal function was re‐tested with the ACTH stimulation test (Synacthen 250 μg, intravenously) and revealed adrenal insufficiency (fasting baseline cortisol levels 4.67 μg/dl [NV 6–18.5]; cortisol peak at 60 minutes: 16.3 μg/dl, [NV > 22 μg/dl]). He was started on glucocorticoid replacement therapy with a satisfactory clinical response.

## 3 DISCUSSION

DS is a rare condition, first described in 1965 in a girl with microcephaly, eczema, and peculiar facial features. She also presented a soft high‐pitched cry, impaired intellectual development, and growth impairment with delayed bone age. No genetic investigation was performed with the exception of karyotype that was normal. Blood cell count and routine urine examination at 7 months were normal. No endocrine test and no brain imaging was performed (Dubowitz, 1965). Since this first case, over 200 cases have been described so far, but no single causative gene has been identified.

The genetics of DS remains an important unsolved and challenging issue. In a recent paper, there have been analyzed various syndromes, such as Dubowitz syndrome, Hallermann‐Streiff syndrome, PHACE syndrome, Oculocerebrocutaneous syndrome, Aicardi syndrome, and others. The authors’ conclusion was that a single gene responsible of DS was not identified due to the wide clinical and molecular heterogeneity (Boycott et al., 2018). Moreover, mosaicisms, epigenetics, interactions between genes and environment, and other non‐Mendelian factors probably may contribute to the phenotype variability. The same hypothesis was made for other rare conditions such as Toriello‐Carey syndrome, Fryns syndrome, PEHO syndrome, and 3C syndrome (Boycott et al., 2018; Carey & Viskochil, 2002). To date, genetic investigations have been performed only in a small percentage (about 10%) of all patients diagnosed as having DS (Innes et al., 2018).

In the current case, aCGH showed a microdeletion in the region 14q 32.31–14q32.33 extended for about 4.6 Mb. To our knowledge, this is the second case of DS reported in the literature with a chromosome deletion in the 14q terminal region detected by aCGH (Darcy et al., 2011). The previous case was a Mexican girl with a terminal deletion at 14q32.33 to 14qter corresponding to genomic coordinates 103,572,825 to 106,339,477 according to hg18 annotation, extended for about 2.77 Mb. This region includes 48 genes, 16 of which have a known phenotype. Comparing the two patients, it emerges that our case exhibits a more extensive deletion of about 4.7 Mb, corresponding to genomic coordinates from 14:102180868 to 14:106850609. This region includes about 222 genes. The different extension of genetic deletions shows some different clinical features, as reported in Table 2. A recent study identified 20 children with 14q terminal deletions, without other genetic anomalies (such as mosaicism, partial trisomies, and ring chromosome 14), and without mention of Dubowitz phenotype (Engels et al., 2012). The same authors tried to describe the phenotype associated with this region alteration (14q32.33). Although the subjects had some phenotypic similarities with our patient, their phenotypes were not suggestive of DS. The endocrine function was not investigated (Engels et al., 2012). MRI of the brain was performed in 3 of 5 reported cases, and it was not described any alteration of the pituitary gland. The growth pattern was variable, and the breakpoint of terminal deletion in 14q chromosome and the size of deletion (expressed in Mb) were different among patients or not reported at all in some cases, thus making impossible to compare these cases with our patient. In a clinical report of 2006, it was described a young girl with 1.6 Mb terminal 14q32.33 deletion, smaller than previously mentioned in the literature, which showed a post‐natal growth retardation without a hypopituitarism on brain MRI—it was described a white matter aspecific hyperintensity—but no GH test or IGF‐1 dosing was performed (Maurin et al., 2006). Since 2006, on nine patients bearing 14q32.3 terminal deletion, only six reported growth retardation without mention about hormonal deficits, no data about delayed puberty or anomalies of pituitary gland on brain RMI. In another clinical report in 2011, a 3 year‐aged boy with a deletion of chromosome 14q32.33 showed a normal growth pattern and a microcystic changes in the pineal gland, without mention of endocrinological anomalies (Holder et al., 2012). No endocrine deficit was mentioned in a 5.5 year‐aged girl with normal auxological parameters, and a deletion proved to be maternally derived and telomeric in 14q32.31, with the proximal breakpoint located between D14S292 (deleted) and D14S985 (not deleted; Karnebeek et al., 2002). In conclusion, focusing on endocrinological features of our patient, growth impairment is well mentioned in the Dubowitz syndrome, while it is inconstant in the 14q terminal deletion syndrome; pituitary gland anomalies on MRI and endocrinological anomalies (hypercortisolism, GHD) are mentioned in DS and not in the 14q terminal deletion syndrome (Hirano et al., 1996; Oguz et al., 2003; Soyer et al., 1995). Since our patient showed endocrinological features described in DS, it should be useful to investigate endocrinological pattern and Dubowitz phenotype in other patients with 14q terminal deletion in order to establish if 14q terminal deletion could be a possible cause of DS.

**TABLE 2:** Comparison of Dubowitz syndrome phenotypical characteristics between our patient and the girl with the same genetic microdeletion

Dubowitz syndrome characteristics (Hansen et al., 1995; Innes et al., 2018; Tsukahara & Opitz, 1996; Vieluf et al., 1990)	Female subject previously described (Darcy et al., 2011)	Male subject presented in this report
Birth		
Mean gestational age >36 weeks	33	38
Mean birth weight >2.2 kg	1,477	2.5
Mean birth length <50 cm	42	45
Low birth OFC (<3°centile)	Yes (30 cm)	Yes (32 cm)
Prenatal growth retardation (<3°P)	Yes	Yes
Postnatal growth retardation	Yes	Yes
Neonatal		
Hypotonia	Yes	Yes
Respiratory problems	Yes	No
Feeding difficulties/GI problems	Yes	Yes
Psychomotor retardation	Yes	Yes
Microcephaly (<3°P)	Yes	Yes
Main clinical features		
High forehead	Yes	Yes
Blepharophimosis	Yes	Yes
Ptosis	Yes	Yes
Epicanthus/telecanthus	Yes	Yes
Downward slanting palpebral fissures	Yes	Yes
Upturned nares	Yes	Yes
Round‐tipped nose	Yes	Yes
Low‐set ears	Yes	Yes
Palate anomalies	No	Yes
Micrognathia	Yes	Yes
Open‐mouth habitus	Yes	Yes
Strabismus	Yes	Yes
Tooth problems	Yes	Yes
Thin hair	No	Yes
Eczema	No	Yes
Clinodactyly of 5th fingers	No	No
Cutaneous syndactyly	No	No
Retarded bone maturation	Not reported	Yes
Congenital heart defects	Not reported	Yes
Cryptorchidism/hypospadias	Female	Yes
Small asymmetrical testis	Female	Yes
Enuresis	Not reported	Yes
Seizures	No	No
High‐pitched voice	Yes	Yes
Hyperactivity	No	No
Developmental disabilities	Yes	Yes
Other secondary features		
Pituitary abnormalities	Not reported	Yes
Endocrinological disorders	Not reported	Yes
Vomiting	Yes	Yes
Chronic diarrhea/stipsis	Not reported	Yes
Allergy	Not reported	Yes
Frequent infections	Not reported	No
Hematological disorders	Not reported	Yes

As genetic assessment in our patient's parents resulted normal, the detected deletion is presumably *de novo*. This finding is in line with the emerging hypothesis of *de novo* genetic variants responsible for DS (Innes et al., 2018). The only previous case of DS reported in the literature with a chromosome deletion in the 14q terminal region detected by aCGH was a Mexican girl, our patient being the first boy with DS and 14q terminal deletion reported so far (Table 2).

The genitourinary anomalies described in our patient are consistent with DS genitourinary alterations occurring in over 50% of male patients, i.e., cryptorchidism, inguinal hernia, hypospadias, small asymmetrical testes, small penis, and hypoplastic genitalia/scrotum (Swartz et al., 2003; Tsukahara & Opitz, 1996). Both patients reported so far with the chromosome deletion in the 14q terminal region detected by aCGH had the most common features of DS such as microcephaly, high forehead, blepharophimosis, ptosis, epicanthus/telecanthus, downward slanting palpebral fissures, upturned nares, round‐tipped nose, low‐set ears, micrognathia, open‐mouth habitus, strabismus, tooth problems, and high‐pitched voice. Differently from the previous female patient, in addition to genitourinary anomalies (bilateral cryptorchidism and small asymmetrical testes), the male patient described here also shows palate malformation with a high‐arched palate, thin hair, and eczematous skin that are well described in the first case of DS and in many subsequent case reports. Another finding common to both patients was pre‐ and postnatal growth failure, but endocrine tests, bone age assessment, and brain imaging were not performed in the Mexican girl. The clinical and laboratory features of our boy suggest that 14q terminal deletion is associated with many features of DS, with additional characteristics not explored in the girl with the same deletion and Dubowitz phenotype.

The review of previous cases suggests that different genetic alterations (chromosome deletions as well as various gene variants) can be associated with Dubowitz phenotype. These findings support the hypothesis that DS could be a mix of disorders with a similar phenotypic expression but a different genotype, instead of being a single syndromic entity (Giordano et al., 2016; Urquhart et al., 2015).

Pre‐ and postnatal growth in children with a sub‐telomeric 14q deletion is variable. Pre‐ or post‐natal growth retardation has been reported in 23% to 38% of children with 14q‐terminal‐deletion (Engels et al., 2012; Karnebeek et al., 2002). Unfortunately, endocrine tests and brain imaging were not systematically performed in the subjects with DS. In our patient, pituitary function tests showed both GH and adrenal insufficiency. Brain MRI revealed a hypoplasia of the anterior pituitary gland and a posterior pituitary ectopically located in the stalk, a picture strongly consistent with hypopituitarism (Arifa et al., 1999; Huber et al., 2011; Mohrenschlager et al., 1998).

A 12‐year‐old boy with postnatal growth restriction and a clinical diagnosis of DS with hypoplastic pituitary gland, hypoplastic stalk, ectopic posterior pituitary, and GH deficiency was previously reported. As genetic analyses were not performed, the relationship between patient's genotype, DS, and pituitary function was not investigated (Oguz et al., 2003). The first documented GH deficiency in a patient with DS was successfully treated with GH appeared in 1996 (Hirano et al., 1996), but brain imaging and phenotype were not reported.

The finding of GH‐deficiency in our case is consistent with the brain MRI picture showing two components of the classical triad associated with hypopituitarism (the third lacking feature being the pituitary stalk agenesis). However, it is possible that GH deficiency was transient as IGF‐1 levels normalized after puberty. An alternative explanation is that the observed subnormal responses of GH to stimulation tests were false positive responses secondary to the delayed puberty. Consistent with this is the spontaneous acceleration of growth rate when the child started puberty. However, testosterone priming was performed prior to the stimulation tests in order to reduce the probability of false positive results.

Nevertheless, the response to GH therapy in the first 6 months was satisfactory. The efficacy of GH therapy in children with DS has not been reported so far, though in a previous out‐of‐date article a vague “positive” response was mentioned (Hirano et al., 1996). Growth hormone treatment was also reported in patients suspected to have DS, in particular two siblings (Dentici et al., 2011) and a boy with a 18p11.23‐p11.31 microduplication (Giordano et al., 2016) who showed a satisfactory increase in height without mention of side effects. Unfortunately, height gain and height velocity were not reported. In a recent study, a patient with DS underwent extensive laboratory and radiological investigations revealing a small pituitary gland and growth impairment without mention on GH‐replacement therapy. Another patient recently diagnosed with DS and with a de novo 3.89 Mb interstitial deletion at chromosome 17q24.2, showed GH deficiency and a normal response to ACTH test, with no evidence of pituitary malformations on brain MRI performed at the age of 4 months. He required GH replacement therapy from the age of 20 months to 13 years (discontinued due to the worsening of kyphosis and scoliosis) (Soyer et al., 1995), without showing alterations of the blood cell count (Stewart et al., 2014). IGF‐1 levels were normal (368 ng/ml) at the age of 16 years and Tanner pubertal stage IV. When our patient developed pancytopenia, we hypothesized it to be related to the syndrome rather than GH therapy, but in agreement with the parents, as a precautionary measure, we decided to stop the treatment. As expected, blood cells did not normalize even 5 years after GH therapy discontinuation. It is well known that the Dubowitz syndrome is characterized by immune defect, increased risk of blood dyscrasia, bone marrow failure, and malignancy. The mild pancytopenia of our patient could be interpreted as the initial sign of a bone marrow dysfunction and should be monitored in the future. A previously reported 41‐year‐old patient with DS underwent bone marrow biopsy because of anemia and leukopenia, showing hypocellular bone marrow with trilineage hypoplasia (Kapoor, 2015b).

Our case showed at the age of 22 years hypergonadotropic hypogonadism with small testicular volume and high FSH levels strongly suggestive of Sertoli cells impairment and likely spared Leydig cell function (testosterone concentrations in the low normal range). A single previous case of a 41 year‐old male patient with DS and hypogonadism (total testosterone <20 ng/dl and small testicular volume, 5 ml) was described previously, but no information about his pubertal progression was reported (Opitz et al., 1973).

GH deficiency may be a feature of DS that requires brain MRI to demonstrate consistent pituitary alterations. GH deficiency may be a “transitory” finding and should be monitored through repeated measurements of IGF‐1levels, evaluation of puberty progression, and eventually re‐assessing GH secretion.

It is noteworthy that this is the first case of DS with adrenal insufficiency reported so far. The pituitary anatomical alterations strongly suggest central adrenal insufficiency. The prompt recognition of adrenal insufficiency is of paramount importance as it is a potential life‐threatening condition if not recognized and adequately treated especially when the patient undergoes stressful events.

## 4 CONCLUSION

We describe a boy with most of DS features associated with14q terminal microdeletion. This association between DS phenotype and 14q terminal abnormality was previously described in one single patient only, so this report provides further support that de novo deletions of this region could give a phenotype evocative of the Dubowitz syndrome. The unique characteristics of our patient were the presence of GH deficiency (with good response to short‐term GH therapy), hypogonadism, and adrenal insufficiency associated with pituitary structural alterations suggestive of hypopituitarism. These findings indicate the need for a strict endocrine monitoring of patients with DS phenotype or the same genotype alterations. Indeed, they can benefit from GH therapy to improve adult height, but especially they should promptly start lifesaving glucocorticoid replacement therapy if adrenal insufficiency is diagnosed.

## ETHICAL COMPLIANCE

The Institutional Review Board of Bambino Gesù Children's Hospital approved this study.

## CONSENT

Written informed consent was obtained from the patient's parents for publication of this case report and any accompanying images.

## CONFLICT OF INTEREST

The authors have nothing to disclose.

## AUTHORS CONTRIBUTIONS

Maria Elisa Amodeo, Elena Inzaghi, and Annalisa Deodati have been involved in analysis and interpretation of data and in drafting the manuscript. Stefano Cianfarani has made substantial contributions to conception and design the study and has given final approval of the version to be published. Elena Inzaghi has agreed to be accountable for all aspects of the work in ensuring that questions related to the accuracy or integrity of any part of the work are appropriately investigated and resolved.

## Data Availability

The data that support the findings of this study are available from the corresponding author upon reasonable request.

## References

[mgg31644-bib-0001] Anderson, M. (1971). Use of the Greulich‐Pyle "Atlas of Skeletal Development of the Hand and Wrist" in a clinical context. American Journal of Physical Anthropology, 35(3), 347–352. 10.1002/ajpa.1330350309 4332699

[mgg31644-bib-0002] Arifa, N. , Leger, J. , Garel, C. , Czernichow, P. , & Hassan, M. (1999). [Cerebral anomalies associated with growth hormone insufficiency in children: Major markers for diagnosis?] Archives De Pediatrie, 6(1), 14–21. 9974090 10.1016/s0929-693x(99)80067-0

[mgg31644-bib-0003] Bedogni, G. , Giannone, G. , Maghnie, M. , Giacomozzi, C. , Di Iorgi, N. , Pedicelli, S. , Peschiaroli, E. , Melioli, G. , Muraca, M. , Cappa, M. , & Cianfarani, S. (2012). Serum insulin‐like growth factor‐I (IGF‐I) reference ranges for chemiluminescence assay in childhood and adolescence. Data from a population of in‐ and out‐patients. Growth Hormone & IGF Research, 22(3–4), 134–138. 10.1016/j.ghir.2012.04.005 22583946

[mgg31644-bib-0004] Boycott, K. M. , Dyment, D. A. , & Innes, A. M. (2018). Unsolved recognizable patterns of human malformation: Challenges and opportunities. American Journal of Medical Genetics. Part C, Seminars in Medical Genetics, 178(4), 382–386. 10.1002/ajmg.c.31665 30580485

[mgg31644-bib-0005] Carey, J. C. , & Viskochil, D. H. (2002). Status of the human malformation map: 2002. American Journal of Medical Genetics, 115(4), 205–220. 10.1002/ajmg.10987 12503116

[mgg31644-bib-0006] Darcy, D. C. , Rosenthal, S. , & Wallerstein, R. J. (2011). Chromosome deletion of 14q32.33 detected by array comparative genomic hybridization in a patient with features of Dubowitz syndrome. Case Reports in Genetics, 2011, 306072. 23074674 10.1155/2011/306072 PMC3447229

[mgg31644-bib-0007] Dentici, M. L. , Mingarelli, R. , & Dallapiccola, B. (2011). The difficult nosology of blepharophimosis‐mental retardation syndromes: Report on two siblings. American Journal of Medical Genetics. Part A, 155A(3), 459–465. 10.1002/ajmg.a.33642 21567902

[mgg31644-bib-0008] Dubowitz, V. (1965). Familial low birthweight dwarfism with an unusual facies and a skin eruption. Journal of Medical Genetics, 2(1), 12–17. 10.1136/jmg.2.1.12 14296916 PMC1012797

[mgg31644-bib-0009] Engels, H. , Schuler, H. M. , Zink, A. M. , Wohlleber, E. , Brockschmidt, A. , Hoischen, A. et al (2012). A phenotype map for 14q32.3 terminal deletions. American Journal of Medical Genetics. Part A, 158A(4), 695–706. 10.1002/ajmg.a.35256 22367666

[mgg31644-bib-0010] Giordano, M. , Muratore, V. , Babu, D. , Meazza, C. , & Bozzola, M. (2016). A 18p11.23‐p11.31 microduplication in a boy with psychomotor delay, cerebellar vermis hypoplasia, chorioretinal coloboma, deafness and GH deficiency. Molecular Cytogenetics, 9, 89. 10.1186/s13039-016-0298-9 27980677 PMC5135744

[mgg31644-bib-0011] Hansen, K. E. , Kirkpatrick, S. J. , & Laxova, R. (1995). Dubowitz syndrome: Long‐term follow‐up of an original patient. American Journal of Medical Genetics, 55(2), 161–164. 7536394 10.1002/ajmg.1320550205

[mgg31644-bib-0012] Hirano, T. , Izumi, I. , & Tamura, K. (1996). Growth hormone deficiency in Dubowitz syndrome. Acta Paediatrica Japonica, 38(3), 267–269. 10.1111/j.1442-200X.1996.tb03484.x 8741320

[mgg31644-bib-0013] Holder, J. L. , Lotze, T. E. , Bacino, C. , & Cheung, S.‐W. (2012). A child with an inherited 0.31 Mb microdeletion of chromosome 14q32.33: Further delineation of a critical region for the 14q32 deletion syndrome. American Journal of Medical Genetics, 158A(8), 1962–1966. 22488736 10.1002/ajmg.a.35289

[mgg31644-bib-0014] Huber, R. S. , Houlihan, D. , & Filter, K. (2011). Dubowitz syndrome: A review and implications for cognitive, behavioral, and psychological features. Journal of Clinical Medicine Research, 3(4), 147–155. 10.4021/jocmr581w 22121397 PMC3194009

[mgg31644-bib-0015] Innes, A. M. , McInnes, B. L. , & Dyment, D. A. (2018). Clinical and genetic heterogeneity in Dubowitz syndrome: Implications for diagnosis, management and further research. American Journal of Medical Genetics. Part C, Seminars in Medical Genetics, 178(4), 387–397. 10.1002/ajmg.c.31661 30580484

[mgg31644-bib-0016] Kapoor, S. (2015). Letter to the editor: Dubowitz syndrome: A unique clinical disorder that is often confused with Bloom syndrome. Journal of Clinical Endocrinology and Metabolism, 100(1), L18–L19. 25559542 10.1210/jc.2014-3931

[mgg31644-bib-0017] Kapoor, S. (2015). Dubowitz syndrome and the increased risk of developing malignancies. Pediatric Allergy and Immunology, 26(8), 820–821. 10.1111/pai.12411 26009798

[mgg31644-bib-0018] Kukushkina, I. P. , Iakubovskii, T. V. , Kudriavtseva, T. V. , & Dmitriev, A. V. (1991). 2 Cases of Dubowitz syndrome in one family. Pediatriia, 2, 80–82. 2057300

[mgg31644-bib-0019] Lyonnet, S. , Schwartz, G. , Gatin, G. , de Prost, Y. , Munnich, A. , & Le Merrer, M. (1992). Blepharophimosis, eczema, and growth and developmental delay in a young adult: Late features of Dubowitz syndrome? Journal of Medical Genetics, 29(1), 68–69. 1552551 10.1136/jmg.29.1.68 PMC1015828

[mgg31644-bib-0020] Marshall, W. A. , & Tanner, J. M. (1970). Variations in the pattern of pubertal changes in boys. Archives of Disease in Childhood, 45(239), 13–23. 10.1136/adc.45.239.13 5440182 PMC2020414

[mgg31644-bib-0021] Martinez, F. J. , Lee, J. H. , Lee, J. E. , Blanco, S. , Nickerson, E. , Gabriel, S. , Frye, M. , Al‐Gazali, L. , & Gleeson, J. G. (2012). Whole exome sequencing identifies a splicing mutation in NSUN2 as a cause of a Dubowitz‐like syndrome. Journal of Medical Genetics, 49(6), 380–385. 22577224 10.1136/jmedgenet-2011-100686 PMC4771841

[mgg31644-bib-0022] Mathieu, M. , Berquin, P. , Epelbaum, S. , Lenaerts, C. , & Piussan, C. (1991). Dubowitz syndrome. A diagnosis not to be missed. Archives Francaises de Pediatrie, 48(10), 715–718. 1793348

[mgg31644-bib-0023] Maurin, M‐l , Brisset, S. , Le Lorc'h, M. , Poncet, V. , Trioche, P. , Aboura, A. , Labrune, P. , & Tachdjian, G. (2006). Terminal 14q32.33 deletion: Genotype–phenotype correlation. American Journal of Medical Genetics, 140A, 2324–2329. 10.1002/ajmg.a.31438 17022077

[mgg31644-bib-0024] Mohrenschlager, M. , Beham, A. , Abeck, D. , & Ring, J. (1998). Atopic eczema in monozygotic twins with Dubowitz syndrome. British Journal of Dermatology, 138(6), 1091–1092. 10.1046/j.1365-2133.1998.02288.x 9747384

[mgg31644-bib-0025] Oguz, K. K. , Ozgen, B. , & Erdem, Z. (2003). Cranial midline abnormalities in Dubowitz syndrome: MR imaging findings. European Radiology, 13(5), 1056–1057. 10.1007/s00330-002-1580-2 12695828

[mgg31644-bib-0026] Opitz, J. M. , Pfeiffer, R. A. , Hermann, J. P. , & Kushnick, T. (1973). Studies of malformation syndromes of man XXIV B: The Dubowitz syndrome. Further Observations. Zeitschrift für Kinderheilkunde, 116, 1–12. 10.1007/BF00438824 4771703

[mgg31644-bib-0027] Soyer, A. D. , & McConnell, J. R. (1995). Progressive scoliosis in Dubowitz syndrome. Spine, 20(21), 2335–2337. 10.1097/00007632-199511000-00012 8553122

[mgg31644-bib-0028] Stewart, D. R. , Pemov, A. , Johnston, J. J. , Sapp, J. C. , Yeager, M. , He, J. , Boland, J. F. , Burdett, L. , Brown, C. , Gatti, R. A. , Alter, B. P. , Biesecker, L. G. , & Savage, S. A. (2014). Dubowitz syndrome is a complex comprised of multiple, genetically distinct and phenotypically overlapping disorders. PLoS One, 9(6), e98686. 10.1371/journal.pone.0098686 24892279 PMC4043752

[mgg31644-bib-0029] Swartz, K. R. , Resnick, D. , Iskandar, B. J. , Wargowski, D. , Brockmeyer, D. , & Opitz, J. (2003). Craniocervical anomalies in Dubowitz syndrome. Three cases and a literature review. Pediatric Neurosurgery, 38(5), 238–243. 10.1159/000069822 12686766

[mgg31644-bib-0030] Thuret, I. , Michel, G. , Philip, N. , Hairion, D. , Capodano, A. M. , & Perrimond, H. (1991). Chromosomal instability in two siblings with Dubowitz syndrome. British Journal of Haematology, 78(1), 124–125. 10.1111/j.1365-2141.1991.tb04395.x 2043468

[mgg31644-bib-0031] Tsukahara, M. , & Opitz, J. M. (1996). Dubowitz syndrome: Review of 141 cases including 36 previously unreported patients. American Journal of Medical Genetics, 63(1), 277–289. 8723121 10.1002/(SICI)1096-8628(19960503)63:1<277::AID-AJMG46>3.0.CO;2-I

[mgg31644-bib-0032] Urquhart, J. E. , Williams, S. G. , Bhaskar, S. S. , Bowers, N. , Clayton‐Smith, J. , & Newman, W. G. (2015). Deletion of 19q13 reveals clinical overlap with Dubowitz syndrome. Journal of Human Genetics, 60(12), 781–785. 10.1038/jhg.2015.111 26377242

[mgg31644-bib-0033] van Karnebeek, C. D. , Quik, S. , Sluijter, S. , Hulsbeek, M. M. , Hoovers, J. M. , & Hennekam, R. C. (2002). Further delineation of the chromosome 14q terminal deletion syndrome. American Journal of Medical Genetics, 110(1), 65–72. 10.1002/ajmg.10207 12116274

[mgg31644-bib-0034] Vieluf, D. , Korting, H. C. , Braun‐Falco, O. , & Walther, J. U. (1990). Dubowitz syndrome: Atopic dermatitis, low birth weight dwarfism and facial dysmorphism. Dermatologica, 180(4), 247–249. 2358105 10.1159/000248040

[mgg31644-bib-0035] Wallerstein, R. , Kacmar, J. , Anderson, C. E. , & Jackson, L. (1997). Dubowitz syndrome in a boy without developmental delay: Further evidence for phenotypic variability. American Journal of Medical Genetics, 68(2), 216–218. 9028461

[mgg31644-bib-0036] Yue, J. , Lu, H. , Lan, S. , Liu, J. , Stein, M. N. , Haffty, B. G. , & Shen, Z. (2013). Identification of the DNA repair defects in a case of Dubowitz syndrome. PLoS One, 8(1), e54389. 23372718 10.1371/journal.pone.0054389 PMC3556036

